# On the Number of Spanning Trees of Graphs

**DOI:** 10.1155/2014/294038

**Published:** 2014-02-10

**Authors:** Ş. Burcu Bozkurt, Durmuş Bozkurt

**Affiliations:** Department of Mathematics, Science Faculty, Selçuk University, Alaeddin Keykubat Campus, 42075 Konya, Turkey

## Abstract

We establish some bounds for the number of spanning trees of connected graphs in terms of the number of vertices (*n*), the number of edges (*m*), maximum vertex degree (Δ_1_), minimum vertex degree (*δ*), first Zagreb index (*M*_1_), and Randić index (*R*_−1_).

## 1. Introduction

Let *G* be a simple connected graph with *n* vertices and *m* edges. Let *V*(*G*) = {*v*_1_, *v*_2_,…, *v*_*n*_} be the vertex set and *E*(*G*) = {*e*_1_, *e*_2_,…, *e*_*m*_} the edge set of *G*. If any two vertices *v*_*i*_ and *v*_*j*_ of *G* are adjacent, that is, *v*_*i*_ *v*_*j*_ ∈ *E*(*G*), then we use the notation *v*_*i*_ ~ *v*_*j*_. For *v*_*i*_ ∈ *V*(*G*), the degree of the vertex *v*_*i*_, denoted by *d*_*i*_, is the number of the vertices adjacent to *v*_*i*_. Let Δ_1_, Δ_2_, and *δ* be the maximum, the second maximum, and the minimum vertex degree of *G*, respectively.

Let *M*_1_ = *M*_1_(*G*) = ∑_*i*=1_^*n*^ *d*_*i*_^2^ be the first Zagreb index [1] and *R*_*α*_ = *R*_*α*_(*G*) = ∑_*v*_*i*_~*v*_*j*__(*d*_*i*_ *d*_*j*_)^*α*^ the general Randić index [2] of the graph *G*, where *α* ≠ 0 is a fixed real number. Note that the Randić index *R*_−1_ = *R*_−1_(*G*) = ∑_*v*_*i*_~*v*_*j*__1/*d*_*i*_ *d*_*j*_ is also well studied in the literature. For more details on *R*_−1_, see [3, 4].

Let *K*_*n*_, *K*_*p*,*q*_  (*p* + *q* = *n*), and *S*_*n*_ denote the complete graph, the complete bipartite graph, and the star graph of order *n*, respectively. Let *G* − *e* be the graph obtained by deleting the edge *e* from the graph *G* and let $\bar{G}$ be the complement of *G*. Let *G*_1_ ∪ *G*_2_ be the vertex-disjoint union of the graphs *G*_1_ and *G*_2_. The graph *G*_1_∨*G*_2_ is obtained from *G*_1_ ∪ *G*_2_ by adding all possible edges from vertices of *G*_1_ to vertices of *G*_2_; that is, $G_{1}\vee G_{2}=\bar{\bar{G_{1}}\cup \bar{G_{2}}}$ [5].

The Laplacian matrix of the graph *G* is the matrix *L*(*G*) = *D*(*G*) − *A*(*G*), where *A*(*G*) and *D*(*G*) are the (0,1)-adjacency matrix and the diagonal matrix of the vertex degrees of *G*, respectively. The normalized Laplacian matrix of *G* is defined as *L* = *D*(*G*)^−1/2^ *L*(*G*)*D*(*G*)^−1/2^, where *D*(*G*)^−1/2^ is the matrix which is obtained by taking (−1/2) power of each entry of *D*(*G*). The Laplacian eigenvalues and the normalized Laplacian eigenvalues of *G* are the eigenvalues of *L*(*G*) and *L*, respectively. Let *μ*_1_ ≥ *μ*_2_ ≥ ⋯≥*μ*_*n*_ be the Laplacian eigenvalues and *λ*_1_ ≥ *λ*_2_ ≥ ⋯≥*λ*_*n*_ the normalized Laplacian eigenvalues of *G*. Note that *μ*_*n*_ = 0, *λ*_*n*_ = 0, and the multiplicities of these zero eigenvalues are equal to the number of connected components of *G*; see [6, 7]. For more details on Laplacian and normalized Laplacian eigenvalues, see [6, 8–10].

The number of spanning trees, *t*(*G*), of the graph *G* is equal to the total number of distinct spanning subgraphs of *G* that are trees. This quantity is also known as the complexity of *G* and given by the following formula in terms of the Laplacian eigenvalues [5]:

(1) $$\begin{array}{c} t\left(G\right)=\frac{1}{n}\overset{n-1}{\underset{i=1}{\prod }}\mu_{i}. \end{array}$$

It is well known that the number of spanning trees of *G* is also expressed by the normalized Laplacian eigenvalues as [5, 6]

(2) $$\begin{array}{c} t\left(G\right)=\left(\frac{\prod_{i=1}^{n}d_{i}}{2m}\right)\overset{n-1}{\underset{i=1}{\prod }}\lambda_{i}. \end{array}$$

Now, we give some known upper bounds on *t*(*G*):

(1) Grimmett [11]:
  (3) $$\begin{array}{c} t\left(G\right)\leq \frac{1}{n}{\left(\frac{2m}{n-1}\right)}^{n-1}, \end{array}$$
(2) Grone and Merris [12]:
  (4) $$\begin{array}{c} t\left(G\right)\leq {\left(\frac{n}{n-1}\right)}^{n-1}\left(\frac{\prod_{i=1}^{n}d_{i}}{2m}\right), \end{array}$$
(3) Nosal [13]: for *r*-regular graphs,
  (5) $$\begin{array}{c} t\left(G\right)\leq n^{n-2}{\left(\frac{r}{n-1}\right)}^{n-1}, \end{array}$$
(4) Cvetković et al. (see [5, page 222]):
  (6) $$\begin{array}{c} t\left(G\right)\leq n^{n-2}{\left(1-\frac{2}{n}\right)}^{\bar{m}}, \end{array}$$
  where $\bar{m}$ is the number of edges of $\bar{G}$,
(5) Das [14]:
  (7) $$\begin{array}{c} t\left(G\right)\leq {\left(\frac{2m-\Delta_{1}-1}{n-2}\right)}^{n-2}, \end{array}$$
(6) Zhang [15]:
  (8) $$\begin{array}{c} t\left(G\right)\leq \left(1+\left(n-2\right)a\right){\left(1-a\right)}^{n-2}\frac{1}{n}{\left(\frac{2m}{n-1}\right)}^{n-1}, \end{array}$$
  where *a* = ((*n*(*n*−1) − 2*m*)/2*mn*(*n*−2))^1/2^,
(7) Feng et al. [16]:
  (9) $$\begin{array}{c} t\left(G\right)\leq \left(\frac{\Delta_{1}+1}{n}\right){\left(\frac{2m-\Delta_{1}-1}{n-2}\right)}^{n-2}, \end{array}$$
  (10) $$\begin{array}{c} t\left(G\right)\leq {\left(\frac{M_{1}+2m-{\left(\Delta_{1}+1\right)}^{2}}{n-2}\right)}^{\left(n-2\right)/2}, \end{array}$$
(8) Li et al. [17]:
  (11) $$\begin{array}{c} t\left(G\right)\leq \delta {\left(\frac{2m-\Delta_{1}-1-\delta }{n-3}\right)}^{n-3}, \end{array}$$
(9) Bozkurt [18]:
  (12) $$\begin{array}{c} t\left(G\right)\leq \left(1+\left(n-2\right)b\right){\left(1-b\right)}^{n-2}{\left(\frac{n}{n-1}\right)}^{n-1}\left(\frac{\prod_{i=1}^{n}d_{i}}{2m}\right), \end{array}$$
  where *b* = ((*n*−1−Δ_1_)/*n*(*n*−2)Δ_1_)^1/2^,
(10) Das et al. [19]:
  (13) $$\begin{array}{c} t\left(G\right)\leq \frac{1}{2m}\Delta_{1}\delta {\left(\frac{2m-\Delta_{1}-\delta }{n-2}\right)}^{n-2}{\left(\frac{n}{n-1}\right)}^{n-1},\\ t\left(G\right)<\frac{1}{4n{\left(n-3\right)}^{n-3}}{\left(\Delta_{1}+\Delta_{2}+1\right)}^{2}{\left(2m-\Delta_{1}-\Delta_{2}-1\right)}^{n-3},\\ t\left(G\right)\leq \frac{1}{n}{\left[\frac{4m^{2}-\left(M_{1}+2m\right)}{\left(n-1\right)\left(n-2\right)}\right]}^{\left(n-1\right)/2}. \end{array}$$

In [11] Grimmet points out that (3) generalizes (5). Grone and Merris [12] observed that, by the application of arithmetic-geometric mean inequality, (4) leads to (3). Das [14] stated that (7) is sharp for *S*_*n*_ or *K*_*n*_, but (3), (4), (5), and (6) are sharp for only *K*_*n*_. In [17] Li et al. indicated that (11) is sharp for *S*_*n*_, *K*_*n*_, *G*≅*K*_1_∨(*K*_1_ ∪ *K*_*n*−2_) or *K*_*n*_ − *e*, but (3) is sharp for only *K*_*n*_ and (7) and (9) are sharp for *S*_*n*_ or *K*_*n*_. However, Das et al. [19] proved that (11) is not true for *K*_*n*_. In [15, 16, 18] the authors showed that (8) is better than (3), (9) is better than (7) and (10), and (12) is better than (4). For more bounds and the relations between the number of spanning trees and the structural parameters of graphs such as connectivity, chromatic number, independence number, and clique number, see [17, 19].

We organize this paper in the following way. In Section 2, we give some previously known results which will be needed later. In Section 3, we obtain some bounds for the number of spanning trees of connected graphs in terms of the number of vertices (*n*), the number of edges (*m*), maximum vertex degree (Δ_1_), minimum vertex degree (*δ*), first Zagreb index (*M*_1_), and Randić index (*R*_−1_). We also showed that some of our results on connected bipartite graphs improve the bounds (9) and (10) for these graphs.

## 2. Lemmas

In this section, we give some useful lemmas which will be used later. Firstly, we introduce an auxiliary quantity for a graph *G* as

(14) $$\begin{array}{c} \alpha =\frac{1}{2}\left[\Delta_{1}+\delta +\sqrt{{\left(\Delta_{1}-\delta \right)}^{2}+4\Delta_{1}}\right], \end{array}$$

where Δ_1_ and *δ* are the maximum and the minimum vertex degree of *G*, respectively.

The result in the following lemma is also known as Kober's inequality.

**Lemma 1 (see [20])**

Let *x*_1_, *x*_2_,…, *x*_*N*_ be nonnegative numbers and let

(15) $$\begin{array}{c} \beta =\frac{1}{N}\overset{N}{\underset{i=1}{\sum }}x_{i},\;\;\gamma ={\left(\overset{N}{\underset{i=1}{\prod }}x_{i}\right)}^{1/N} \end{array}$$

be their arithmetic and geometric means, respectively. Then

(16) $$\begin{array}{c} \frac{1}{N\left(N-1\right)}\underset{i<j}{\sum }{\left(\sqrt{x_{i}}-\sqrt{x_{j}}\right)}^{2}\leq \beta -\gamma \leq \frac{1}{N}\underset{i<j}{\sum }{\left(\sqrt{x_{i}}-\sqrt{x_{j}}\right)}^{2}. \end{array}$$

Moreover, equality in (16) holds if and only if *x*_1_ = *x*_2_ = ⋯ = *x*_*N*_.

**Lemma 2 (see [21])**

Let *G* be a graph with *n* vertices and normalized Laplacian matrix *L* without isolated vertices. Then

(17) $$\begin{array}{c} \overset{n}{\underset{i=1}{\sum }}\lambda_{i}=\mathrm{tr}\left(L\right)=n,\\ \overset{n}{\underset{i=1}{\sum }}\lambda_{i}^{2}=\mathrm{tr}\left(L^{2}\right)=n+2\sum_{v_{i}\sim v_{j}}\frac{1}{d_{i}d_{j}}. \end{array}$$

**Lemma 3 (see [8])**

Let *G* be a graph with *n* vertices and without isolated vertices. Then *λ*_1_ = *λ*_2_ = ⋯ = *λ*_*n*−1_ if and only if *G* is a complete graph *K*_*n*_.

**Lemma 4 (see [8])**

Let *G* be a connected graph with *n* > 2 vertices. Then *λ*_2_ = *λ*_3_ = ⋯ = *λ*_*n*−1_ if and only if *G*≅*K*_*n*_ or *G*≅*K*_*p*,*q*_.

Note that, the Laplacian eigenvalues of a bipartite graph *G* coincide with its signless Laplacian eigenvalues, that is, eigenvalues of the signless Laplacian matrix *D*(*G*) + *A*(*G*) [9, 10, 22]. Thus, one can arrive at the following result.

**Lemma 5 (see [23, 24])**

Let *G* be a connected bipartite graph with *n* ≥ 3 vertices and let Δ_1_ be the maximum vertex degree of *G*. Then

(18) $$\begin{array}{c} \mu_{1}\geq \alpha \geq \Delta_{1}+1 \end{array}$$

with either equalities if and only if *G* is a star graph *S*_*n*_.

**Lemma 6 (see [9])**

Let *G* be a graph with *n* vertices. Then *μ*_1_ ≤ *n*, with equality if and only if $\bar{G}$ is disconnected.

**Lemma 7 (see [14])**

Let *G* be a connected graph with *n* ≥ 3 vertices. Then *μ*_2_ = *μ*_3_ = ⋯ = *μ*_*n*−1_ if and only if *G*≅*K*_*n*_ or *G*≅*S*_*n*_ or *G*≅*K*_Δ_1_,Δ_1__.

## 3. Main Results

Recently, Das et al. [19] established upper and lower bounds on *t*(*G*) applying Kober's inequality to Laplacian eigenvalues of a connected graph *G*. We now consider Kober's inequality for the normalized Laplacian eigenvalues of *G* in order to present some bounds on *t*(*G*).

**Theorem 8**

Let *G* be a connected graph with *n* vertices, *m* edges, and Randić index *R*_−1_. Then

(19) t(G)≤(∏i=1ndi2m) ×[1(n−1)(n−2)(n2−(n+2R−1))](n−1)/2,

(20) t(G)≥(∏i=1ndi2m) ×[1(n−1)(n2−(n−2)(n+2R−1))](n−1)/2.

Moreover, equalities in (19) and (20) hold if and only if *G*≅*K*_*n*_.

**Proof**

Taking *N* = *n* − 1, *x*_*i*_ = *λ*_*i*_^2^, and *i* = 1,2,…, *n* − 1 in Lemma 1, we get

(21) ∑i<j(λi−λj)2(n−1)(n−2)≤∑i=1n−1λi2n−1−(∏i=1n−1λi)2/(n−1)≤∑i<j(λi−λj)2n−1.

By the proof of Theorem 7 in [19] and Lemma 2, we have

(22) ∑i<j(λi−λj)2=(n−1)∑i=1n−1λi2−(∑i=1n−1λi)2=(n−1)(n+2∑vi~vj1didj)−n2=(n−1)(n+2R−1)−n2.

Then, combining (21) with this and (2), we get

(23) (n−1)(n+2R−1)−n2(n−1)(n−2)≤n+2R−1n−1−(2mt(G)∏i=1ndi)2/(n−1)≤(n−1)(n+2R−1)−n2n−1.

This implies that

(24) $$\begin{array}{c} {\left(\frac{2mt\left(G\right)}{\prod_{i=1}^{n}d_{i}}\right)}^{2/(n-1)}\leq \frac{1}{\left(n-1\right)\left(n-2\right)}\left(n^{2}-\left(n+2R_{-1}\right)\right),\\ {\left(\frac{2mt\left(G\right)}{\prod_{i=1}^{n}d_{i}}\right)}^{2/(n-1)}\geq \frac{n^{2}}{n-1}-\left(\frac{n-2}{n-1}\right)\left(n+2R_{-1}\right). \end{array}$$

Hence we obtain the first part of the theorem. Now we suppose that the equalities in (19) and (20) hold. Then, by Lemma 1, we have *λ*_1_ = *λ*_2_ = ⋯ = *λ*_*n*−1_. Therefore, from Lemma 3, we get that *G*≅*K*_*n*_.

Conversely, we can easily see that the equalities in (19) and (20) hold for the complete graph *K*_*n*_.

We now consider the above theorem for connected bipartite graphs.

**Theorem 9**

Let *G* be a connected bipartite graph with *n* > 2 vertices, *m* edges, and Randić index *R*_−1_. Then

(25) t(G)≤(∏i=1ndim) ×[1(n−2)(n−3)((n−2)2−(n+2R−1−4))](n−2)/2,t(G)≥(∏i=1ndim) ×[(n−2)−(n−3n−2)(n+2R−1−4)](n−2)/2.

Moreover, equalities in (25) hold if and only if *G*≅*K*_*p*,*q*_.

**Proof**

Taking *N* = *n* − 2, *x*_*i*_ = *λ*_*i*_^2^, and *i* = 2,…, *n* − 1 in Lemma 1, we have

(26) ∑2≤i<j≤n−1(λi−λj)2(n−2)(n−3)≤∑i=2n−1λi2n−2−(∏i=2n−1λi)2/(n−2)≤∑2≤i<j≤n−1(λi−λj)2n−2.

Since *G* is bipartite, we also have *λ*_1_ = 2 [6]. Then, by Lemma 2, we get

(27) ∑2≤i<j≤n−1(λi−λj)2 =(n−2)∑i=2n−1λi2−(∑i=2n−1λi)2 =(n−2)(n+2∑vi~vj1didj−4)−(n−2)2 =(n−2)(n+2R−1−4)−(n−2)2.

Therefore, combining (26) with this and (2), we arrive at

(28) (n−2)(n+2R−1−4)−(n−2)2(n−2)(n−3)  ≤n+2R−1−4n−2−(mt(G)∏i=1ndi)2/(n−2)  ≤(n−2)(n+2R−1−4)−(n−2)2n−2.

This implies that

(29) (mt(G)∏i=1ndi)2/(n−2) ≤1(n−2)(n−3)((n−2)2−(n+2R−1−4)),(mt(G)∏i=1ndi)2/(n−2)≥(n−2)−(n−3n−2)(n+2R−1−4).

Hence we get the inequalities (25). Now we suppose that the equalities in (25) hold. Then, by Lemma 1, we have *λ*_2_ = *λ*_3_ = ⋯ = *λ*_*n*−1_. Therefore, by Lemma 4, we conclude that *G*≅*K*_*p*,*q*_.

Conversely, we can easily see that the equalities in (25) hold for the complete bipartite graph *K*_*p*,*q*_.

We now present the improvement of the results obtained in [16] for bipartite graphs.

**Theorem 10**

Let *G* be a connected bipartite graph with *n* ≥ 3 vertices and *m* edges and let *α* be given by (14). Then

(30) $$\begin{array}{c} t\left(G\right)\leq \left(\frac{\alpha }{n}\right){\left(\frac{2m-\alpha }{n-2}\right)}^{n-2} \end{array}$$

with equality if and only if *G*≅*S*_*n*_.

**Proof**

From (1) and Lemmas 5–7, one can prove (30) in a similar way to the proof of Theorem 1.1 in [16].

**Remark 11**

From Lemma 5, we have *μ*_1_ ≥ *α* ≥ Δ_1_ + 1. Then by the proof of Theorem 1.1 in [16], one may conclude that (30) improves (9) for bipartite graphs.

**Theorem 12**

Let *G* be a connected bipartite graph with *n* ≥ 3 vertices, *m* edges, and first Zagreb index *M*_1_ and let *α* be given by (14). Then

(31) $$\begin{array}{c} t\left(G\right)\leq {\left(\frac{M_{1}+2m-\alpha^{2}}{n-2}\right)}^{\left(n-2\right)/2} \end{array}$$

with equality if and only if *G*≅*S*_*n*_.

**Proof**

From (1) and Lemmas 5–7, the proof of (31) can be easily given in a similar way to the proof of Theorem 1.2 in [16].

**Remark 13**

From Lemma 5, we have *μ*_1_ ≥ *α* ≥ Δ_1_ + 1. Then by the proof of Theorem 1.2 in [16], one may conclude that (31) improves (10) for bipartite graphs.

**Remark 14**

By using the similar manner in [16], one can easily show that (30) is better than (31). Moreover, if we can obtain a new bound *μ*_1_ ≥ *α*′ ≥ *α* ≥ Δ_1_ + 1, then we can improve the bounds (30) and (31).

**Example 15**

Let *G* be a graph with vertex set *V*(*G*) = {*v*_1_, *v*_2_, *v*_3_, *v*_4_, *v*_5_} and edge set

(32) E(G)={e1=v1v2,e2=v1v3,  e3=v2v4,e4=v2v5,e5=v4v5}.

For this graph, *t*(*G*) is equal to 3. At rounded three decimal places, the bounds (8), (9), (11), (12), (13), and (19) give *t*(*G*) ≤ 5.659, *t*(*G*) ≤ 6.400, *t*(*G*) ≤ 6.250, *t*(*G*) ≤ 5.224, *t*(*G*) ≤ 5.859, *t*(*G*) ≤ 7.200, *t*(*G*) ≤ 6.422, and *t*(*G*) ≤ 5.104, respectively. This shows that the bound (19) is the best among the mentioned upper bounds for *t*(*G*). But in general sense, they are not comparable.
